# Transcriptome and comparative gene expression analysis of *Phyllostachys edulis* in response to high light

**DOI:** 10.1186/s12870-016-0720-9

**Published:** 2016-01-28

**Authors:** Hansheng Zhao, Yongfeng Lou, Huayu Sun, Lichao Li, Lili Wang, Lili Dong, Zhimin Gao

**Affiliations:** State Forestry Administration Key Open Laboratory on the Science and Technology of Bamboo and Rattan, Beijing, 100102 China; Institute of Gene Science for Bamboo and Rattan Resources, International Center for Bamboo and Rattan, Beijing, 100102 China

**Keywords:** Moso bamboo, RNA-Seq, Photosynthesis, Gene expression, Transcript factors

## Abstract

**Background:**

Photosynthesis plays a vital role as an energy source for plant metabolism, and its efficiency may be drastically reduced owing to abiotic stresses. Moso bamboo (*Phyllostachys edulis*), is a renewable and versatile resource with significant ecological and economic value, which encounters high light stress with large amplitude in natural environment. However, the gene expression profiles in response to high light were elusive in bamboo.

**Results:**

We firstly performed physiological experiments on moso bamboo leaves treated with high light (1200 μmol · m^−2^ · s^−1^). Based on the physiological results, three samples of leaves treated with high light for 0 h (CK), 0.5 h (0.5H), and 8 h (8H) were selected to perform further high-throughput RNA sequencing (RNA-Seq), respectively. Then, the transcriptomic result demonstrated that the most genes were expressed at a statistically significant value (FPKM ≥ 1) and the RNA-Seq data were validated via quantitative real time PCR. Moreover, some significant gene expression changes were detected. For instance, 154 differentially expressed genes were detected in 0.5H *vs*. CK, those in 8H *vs*. CK were 710, and 429 differentially expressed genes were also identified in 0.5H *vs*.8 H. Besides, 47 gene annotations closely related to photosynthesis were refined, including 35 genes annotated as light-harvesting chlorophyll a/b-binding (LHC) proteins, 9 LHC-like proteins and 3 PsbSs. Furthermore, the pathway of reactive oxygen species (ROS) in photosynthesis was further analyzed. A total of 171 genes associated with ROS-scavenging were identified. Some up-regulated transcript factors, such as NAC, WRKY, AR2/ERF, and bHLH, mainly concentrated in short-term response, while C_2_H_2_, HSF, bZIP, and MYB were largely involved in short and middle terms response to high light.

**Conclusion:**

Based on the gene expression analysis of moso bamboo in response to high light, we thus identified the global gene expression patterns, refined the annotations of LHC protein, LHC-like protein and PsbS, detected the pathway of ROS as well as identified ROS-scavenging genes and transcription factors in the regulation of photosynthetic and related metabolisms. These findings maybe provide a starting point to interpret the molecular mechanism of photosynthesis in moso bamboo under high light stress.

**Electronic supplementary material:**

The online version of this article (doi:10.1186/s12870-016-0720-9) contains supplementary material, which is available to authorized users.

## Background

The woody bamboo classified in the grass family Poaceae, Bambusoideae, tribe Bambusease, was considered as one of the most important non-timber forest resources in the world. In the recent years, the woody bamboo had received much attention in the ecological and economic aspects, since it has diverse advantages, such as fast-growth, high strength-to-weight ratio, strongly lignified culms, and strongly carbon fixation capability. The woody bamboo is one of the best agents for carbon sequestration in the subtropical areas of China, which is 2 to 4 times more effective than Chinese fir and pines [1]. Photosynthesis plays essential roles in supplying carbon-hydrates for the exhibition of bamboo characteristics. However, the study on spectroscopic features, capacity of forming homotrimers and structural stabilities of different bamboo isoforms (Lhcb1-3) showed that they possess similar characteristics as those in other higher plants in spite of small differences [2], which means that bamboo may have a special mechanism in the processes of light utilization and regulation for its fast growth though it is unknown.

The comprehensive gene expression profiles of bamboo involved in photosynthesis are significant to understand the molecular basis and dynamic gene expression in response to high light. As one of essential next-generation sequencing technology, the high-throughput RNA sequencing (RNA-Seq) is capable to reveal a snapshot of RNA presence and quantity from a genome at a given moment in time [3, 4]. Relying on the accomplishment of the draft genome sequence of moso bamboo [5], RNA-Seq data will help reasonably interpret the functional elements of the genome and reveal the molecular composition under light stress. Previous studies of expression profiles mainly focused on different tissues [6–9]. To date, the genome-wide expression profile of photosynthesis-related genes in response to high light still remains elusive.

To provide a genome-wide insight into the molecular and regulated mechanism in response to high light, the Chinese endemic bamboo species, moso bamboo (*Phyllostachys edulis*) was focused in further analysis. Based on the analysis of photosynthetic physiology, three samples including leaves treated with high light (1200 μmol · m^−2^ · s^−1^) for 0 h (CK), 0.5 h (0.5H) and 8 h (8H) were used for RNA isolation, respectively. We identified a large number of expressed genes in deeply sequencing pool based on RNA-Seq data from the three samples using the Illumina HiSeq 2000 sequencing platform. The further analysis of gene clustering, gene expression patterns, differentially expressed genes and transcript factors was conducted, the results facilitated our understanding of the photosynthesis, reactive oxygen species (ROS), and non-photochemical quenching (NPQ) in response to high light. This maybe provide a resource of expression profiles for further experimental design as well as serve as a foundation for further studies on function of genes and regulated network under light stress, particularly the transcript factors involved in response to high light.

## Results and discussion

### Photosynthetic physiology analysis of bamboo

**Fig. 1:**
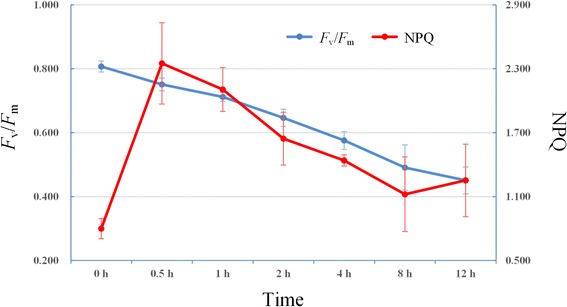
Distribution of NPQ kinetics and *F*_v_/*F*_m_. X-axes represented light time. Error bars indicate standard deviation in NPQ kinetics and *F*_v_/*F*_m_

The chlorophyll fluorescence kinetics technique is referred to as a quick and nonintrusive probe in the studies of plant photosynthetic function. Among the fluorescence parameters, NPQ kinetics is frequently used as a tool to characterize the non-photochemical quenching processes, and the maximal photochemical efficiency (*F*_v_/*F*_m_) is an index to estimate the degree of photoinhibition [10]. Therefore, NPQ kinetics and *F*_v_/*F*_m_ were investigated in moso bamboo leaves under high light (1200 μmol · m^−2^ · s^−1^) for up to 12 h, respectively. Thus, the results in Fig. 1 depicted the distribution of *F*_v_/*F*_m_ and NPQ in moso bamboo leaves based on treatments of the same light intensity at different time. The maximal *F*_v_/*F*_m_ appeared in 0 h, then it decreased almost linearly with the increased time under high light. The value of *F*_v_/*F*_m_ at 12 h was decreased by ~ 44.11 % compared to the control (0 h). These indicated that photoinhibition under high light was targeted in moso bamboo leaves, and the degree had constantly enhanced with the increased time of high light. Similarly, NPQ was activated by high light and increased rapidly during the first 0.5 h, and then decreased slowly, finally tended to be stable after 8 h. Taken together, we selected three representative samples, including 0 h (CK), 0.5 h (0.5H) and 8 h (8H), to further perform a series of transcriptomic analysis.

### Overview of the bamboo transcriptome and validation of RNA-Seq data by qRT-PCR

In view of natural daily stress of high light less than 8 hours, three RNA libraries of moso bamboo leaves were selected on the basis of photosynthetic physiological experiments. These libraries were constructed and then pair-end sequenced based on Illumina Highseq-2000 in order to help comprehensively understand a global atlas of the transcriptome in response to high light. After preprocessing and quality control for raw data of RNA-Seq, the clean reads were aligned to the reference genome sequence from Bamboo Genome Database [11] (www.bamboogdb.org, version 1) to estimate the profile of expressed genes in each library. The software of TopHat was employed and core parameters were set based on transcriptome feature and genomic architecture. As shown in Additional file 1, about 321 million reads (~32 Gb) high quality reads, with an average of 107 million reads (~10 Gb) per sample, were finally acquired as all clean reads. Approximately 75.04 % and 6.76 % of total reads were considered as unique reads and multi-position reads, which represented the number of reads mapped to the reference genome with unique position and multi-position, respectively. Because multi-position reads will eventually map into one position of reference genome randomly based the complexity of reference genome as well as the limitation of sequencing and alignment methods, it inevitably has some biases in the analysis of gene expression level. The result of more unique reads and less multi-position reads in our study, therefore, will contribute to produce more reliable alignment data to facilitate the follow-up expression analysis.

**Fig. 2:**
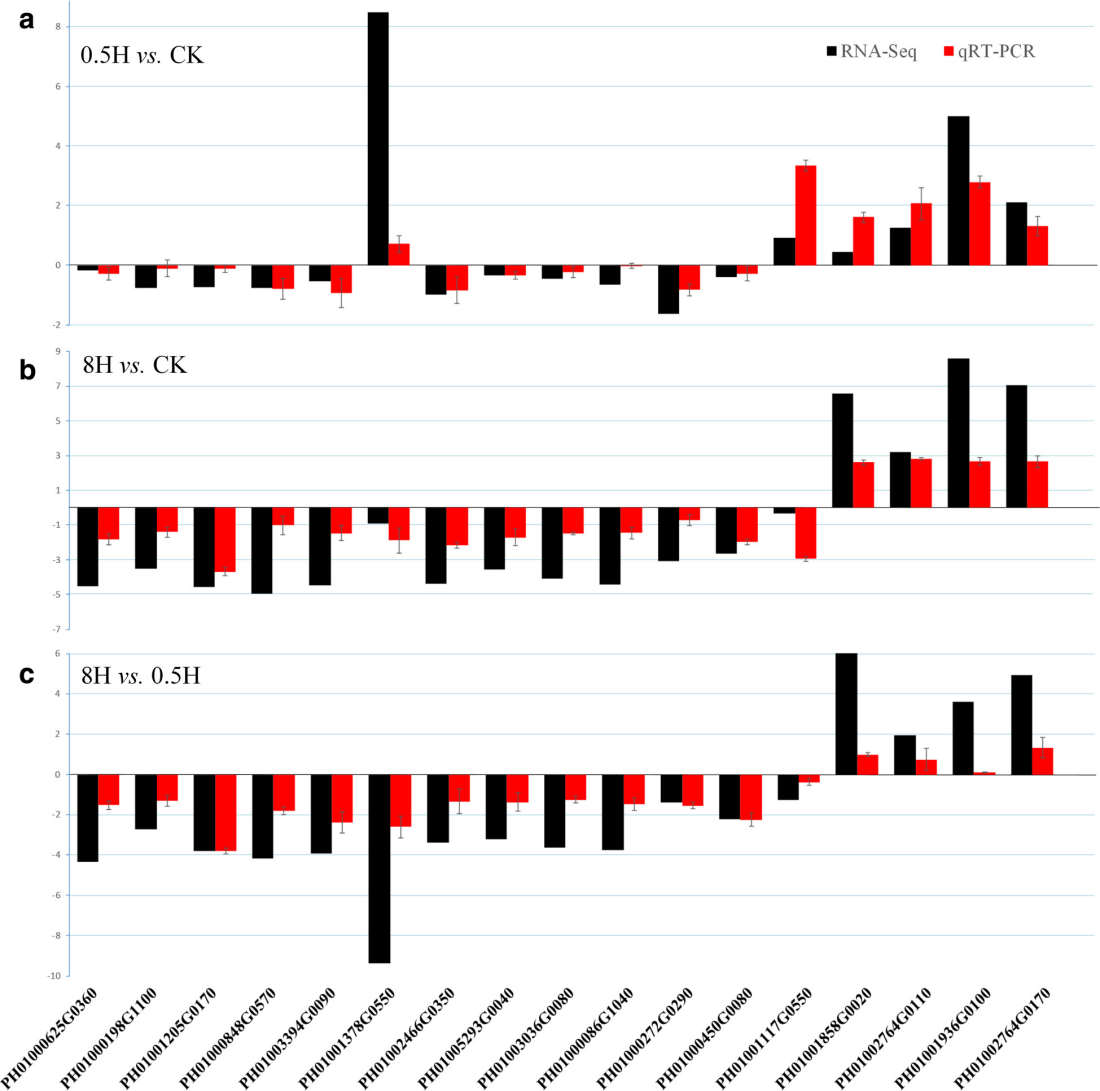
Comparison of relative expression of 17 selected genes based on RNA-Seq data and qRT-PCR data. A histogram of gene expression combined RNA-Seq data and qRT-PCR. X-axes represented 17 selected genes randomly. Y-axes represented log_2_ (relative expression). **a** 0.5H *vs*. CK; **b** 8H *vs*. CK; **c** 8H *vs*.0.5H. Error bars indicate standard deviation in qRT-PCR data

To properly verify the expressed genes based on RNA-Seq, qRT-PCR assays were performed using independently collected samples, which were in the same developmental stage as those used for the RNA-Seq analysis. We selected 17 genes from a larger number of genes associated with photosynthesis. These contained 14 genes belonging to light-harvesting chlorophyll a/b binding (LHC) protein superfamily (10 genes encoding LHC proteins and 4 genes encoding early light-induced proteins) and 3 genes of aquaporin protein family possibly involving in the regulation of stomatal numbers and sizes. Based on validating a subset of RNA-Seq by qRT-PCR, the comparative results of Fig. 2 demonstrated similar expression patterns between RNA-Seq and qRT-PCR, which proved the reliable of RNA-Seq data. Detailed results appeared in Additional file 2.

### Analyzing of expressed genes in bamboo

**Fig. 3:**
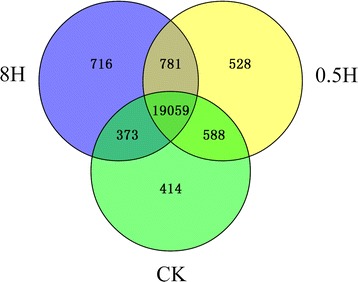
Venn diagram of expressed genes with FPKM ≥ 1 in three samples. There was 20,434, 20,956, and 20,929 expressed genes with FPKM ≥ 1 in CK, 0.5H and 8H, respectively. The number of 19,059 expressed genes in three samples. The number of 781 expressed genes between 0.5H and 8H. The number of 588 expressed genes between CK and 0.5H. The number of 373 expressed genes between CK and 8H. The number of 414 expressed genes include exclusively in CK. The number of 528 expressed genes included exclusively in 0.5H. The number of 716 expressed genes included exclusively in 8H. Detailed information of expressed gene in Venn diagram was attached in Additional file 3

FPKM, also known as Fragments Per Kilobase of gene per Million mapped fragments, was widely utilized in RNA-Seq analysis, aiming to quantify analysis of gene expression levels. To determine which genes were expressed in each sample, the statistic in the distribution of gene expression values was fulfilled among the three samples (Fig. 3 and Additional file 3). The results revealed that all genes in the three libraries of moso bamboo shared similar distribution of gene expression (Additional file 4). Besides, the genes with FPKM > 0 accounted for ~90 % genes of the total annotated genes as well as the number of genes with moderate expression values (1 < FPKM ≤ 100) and high expression values (FPKM >100) accounted for ~68 % of total annotated genes. However, approximately 22 % of the expressed genes were considered as low expression values (0 < FPKM ≤ 1).

Moreover, to explore the conservatively biological function for 19,059 expressed genes in within individual sample (marked as within-sample), the enrichment analysis of Gene Ontology (GO) terms was performed using all bamboo genes as the background (Additional file 5). In total, 131 GO terms, “biological process” (80), “molecular function” (22), and “cellular component” (29), were detected as significant GO terms with adjusted *p*-value <0.01. The results of “biological process” terms illustrated that these expressed genes were highly enriched in the processes associated with “translation (GO:0006412)”, “organ nitrogen compound metabolic process (GO:1901564)”, and “small molecule metabolic process (GO:0044281)”. In the “molecular function” terms, “structural constituent of ribosome (GO:0003735)” and “RNA binding (GO:0003723)” were mainly enriched. Ultimately, some enrichment GO terms in the “cellular component” involved in “cytoplasm (GO:0005737)”, “cytoplasmic part (GO:0044444)” and “ribonucleoprotein complex (GO:0030529)”.

### Clustering affinity search reveals dynamic changes of expressed genes in three samples

**Fig. 4:**
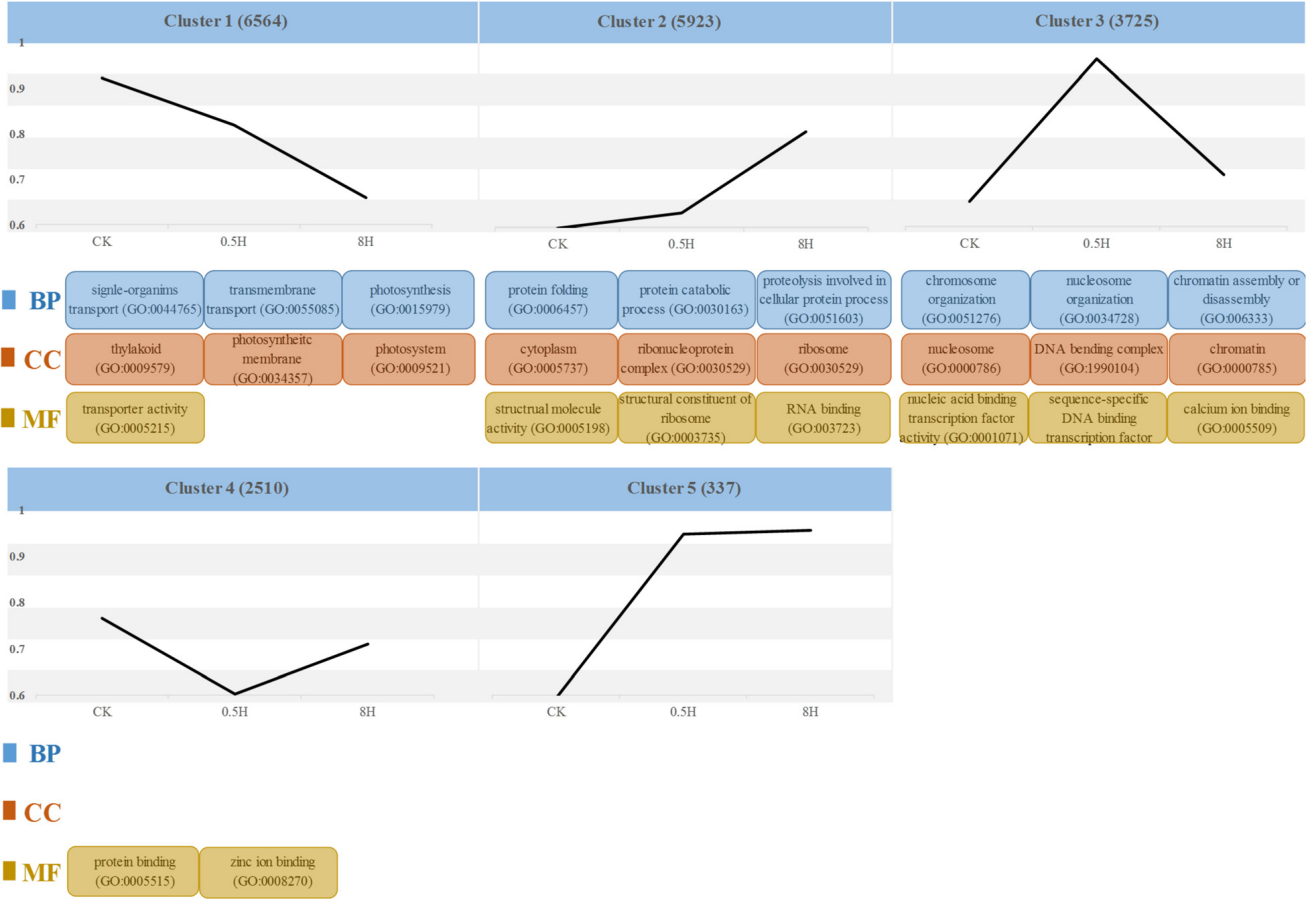
Cluster analysis of expressed genes in moso bamboo. The five groups were identified via the average value of log_2_ (FPKM + 1). The number of gene in each groups was showed in bracketed. Significantly GO terms were depicted based on three GO categories, BP: biological process, CC: cellular component, and MF: molecular function

The clustering affinity search technique (CAST) was broadly applied to elucidating dynamic changes in the transcriptome during different samples [12]. The clustering results utilized by CAST in this study showed 19,059 expressed genes in within-sample were clustered into 5 groups, with the gene numbers within clusters ranging from 337 to 6564. As shown in Fig. 4, five groups of expressed genes shared differentially expressed patterns according to the cluster analysis results. The same pattern contained similar trend of expressed genes, indicating that these genes maybe participate in similar or related biological process. As the biggest group, cluster 1 was of most interest one because a large number of genes associated with photosynthesis were detected, such as 26 genes of chlorophyll a/b binding protein and 16 genes involved in photosystem. The result indicated that the number of genes associated with photosynthesis in cluster 1 were more than other clusters, suggesting these genes maybe play crucial roles in response to high light.

Besides, to better understand and unveil expression characteristics of clustering genes, the analysis of GO terms enrichment was employed. For example, the gene expression in cluster 1 was decreased continuously with the increasing time of light treatment between 0.5H and 8H. GO enrichment also illustrated the terms of “photosynthesis (GO:0015979)”, “photosystem (GO:0009521)” and “transporter activity (GO:0005215)” were enrichment in cluster 1. On the contrary, the gene expression was increased continuously between 0.5H and 8H with the increased light time. The mainly significant GO terms, such as “protein catabolic process (GO:0030163)”, “RNA binding (GO:003723)”, and “ribosome (GO:0030529)”, were enrichment in cluster 2. Compared with CK in the cluster 3, similar expression level appeared in 8H, prior to increased expression level between 0.5H and CK. Major significant GO terms in molecular function, “nucleic acid binding transcription factor activity (GO:0001071)”, “sequence-specific DNA binding transcription factor (GO:0003700)” and “calcium ion binding (GO:0005509)”, indicating some TFs and calcium maybe participate in this process. In addition, since a few data addressed the criteria, a few or none of significant GO terms were identified in cluster 4 and 5. The lists of genes and significant GO terms in each group were stored in Additional file 6.

### Analyzing of differentially expressed genes in three samples

**Fig. 5:**
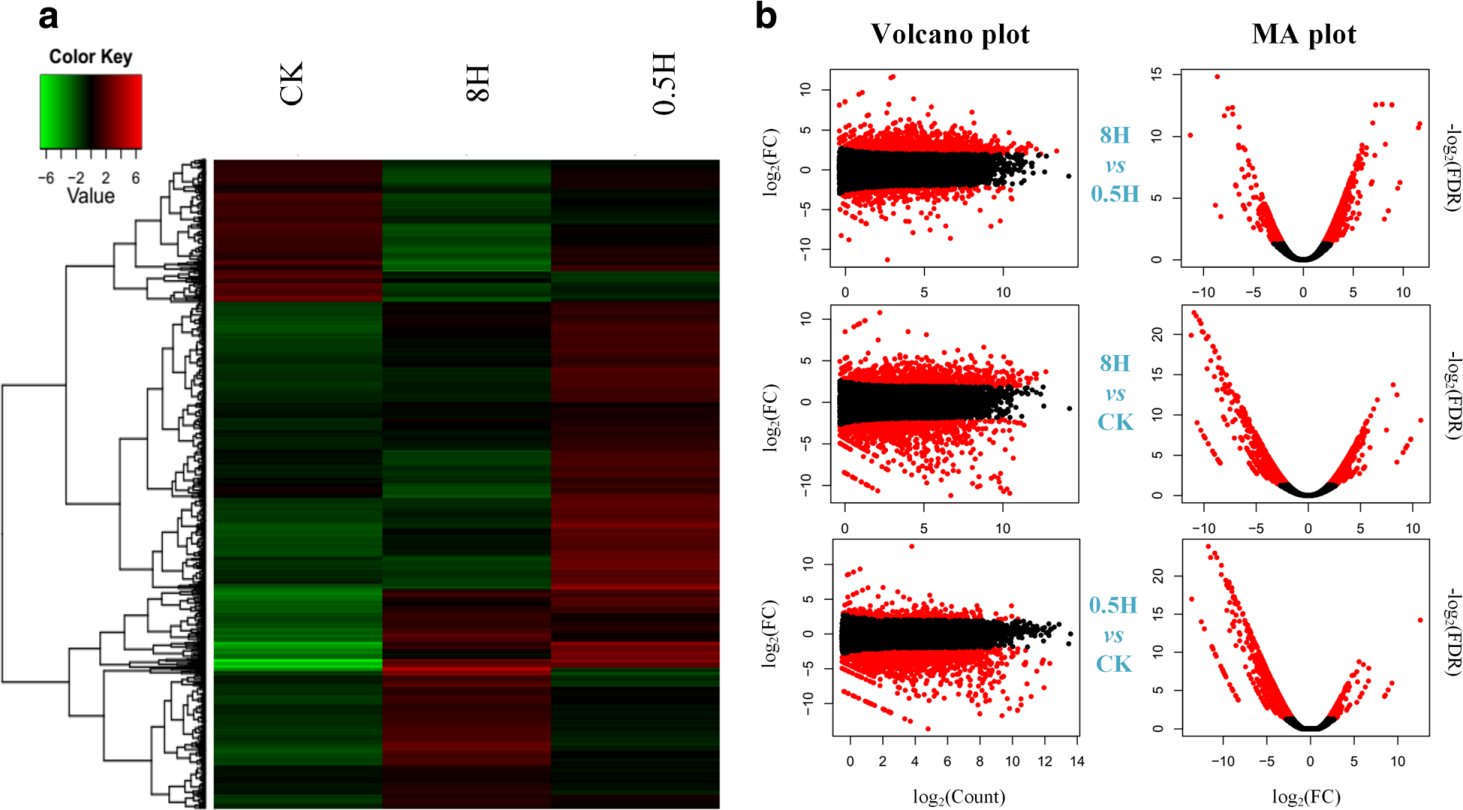
The statistics of differential expression genes. **a** The heatmap based on the log_2_ of FPKM for each gene used for hierarchical analysis at each sample. **b** MA and volcano plots of significant expressed genes for a pair of samples, in 8H *vs*. 0.5H, 8H *vs*. CK and 0.5H *vs*. CK, respectively. Red dot: significant expression, black dot: no significant expression, FDR: false discovery rate, FC: fold change

According into the pair-wise comparison between samples, 1,293 differentially expressed genes (DEGs) were identified utilizing the following cutoff: log_2_FC ≥ 2 or ≤ −2, FDR < 0.01 (Table 1). The number of 154 genes that differed in 0.5H *vs*. CK, included 132 up-regulated genes and 22 down-regulated genes. The number of 710 genes that differed in 8H *vs*. CK, composed of 435 up-regulated genes and 275 down-regulated genes. Ultimately, of the 429 genes that differed in 0.5H *vs*. 8H, 337 genes were up-regulated and 92 genes were down-regulated. Consequently, to vividly illustrate the expression profiles in the identified 1,293 DEGs, the heatmap and plot were drawn in Fig. 5.

**Table 1:** Summary of differentially expressed genes in 0.5H, 8H and CK

Samples	Up-regulated genes	Down-regulated genes
0.5H *vs*. CK	132	22
8H *vs*. CK	435	275
0.5H *vs*. 8H	337	92

**Fig. 6:**
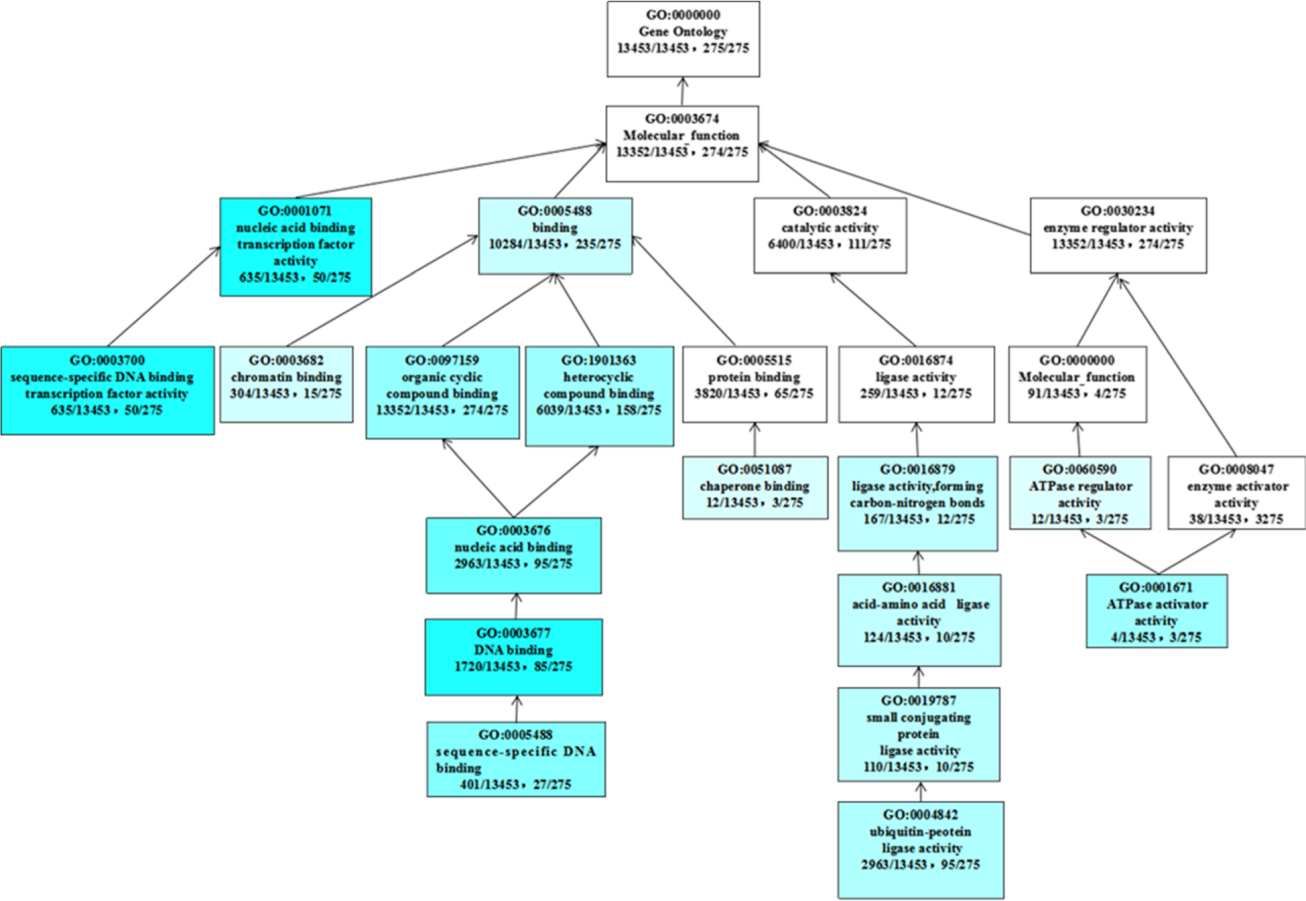
Significant molecular functional terms for the up-regulated genes in 0.5H *vs*. CK. The GO terms were analyzed using an adjusted FDR value ≤0.01 as the cutoff for significant GO categories. The deeper the color, the less the value of adjusted FDR

Besides, we fulfilled GO enrichment analysis to investigate the functional distribution in differentially expressed genes (Additional file 7). The results revealed some significant GO terms with similar function were concentered in certain datasets. For example, GO terms related to transcription factors, including “nucleic acid binding transcription factor activity (GO:0001071)” and “sequence-specific DNA binding transcription factor activity (GO:0003700)”, were enriched in the dataset of DGEs in 0.5H *vs*. CK (Fig. 6). Another example was that 17 significant GO terms, accounting for more than 50 % of the total, were involved in photosystems and related pathways in the dataset of down-regulated DGEs in 8H *vs*. CK, such as “photosystem I (GO:0009522)”, “photosystem (GO:0009521)”, “thylakoid (GO:0009579) and “photosynthesis (GO:0015979)”.

### Identification and analysis of the LHC protein family in bamboo

Photosynthesis provides chemical energy for almost all life on earth. The primary event in photosynthesis involves the absorption of solar energy from sunlight to create electronic excitations in the peripheral antenna of photosynthetic systems and the subsequent transfer of the excitations to a reaction center [13]. An efficient light-harvesting step is critical for the success of photosynthesis. In addition, the LHC proteins, also known as light-harvesting antenna, are the centerpiece of eukaryotic photosynthesis and comprise of the LHC family and several families associated with phtotoprotection, such as the three-helix early light-inducible proteins (ELIPs), two-helix stress-enhanced proteins (SEPs), one-helix light-inducible proteins (OHPs), and the photosystem II subunit S (PsbS) [14, 15]. Based on genome-wide analysis, the LHC proteins and ELIPs in *Arabidopsis thaliana* and *Oryza sativa* were analyzed [16]. However, the genome-wide study of LHC proteins was still unavailable in bamboo. We identified and refined the LHC protein superfamily in moso bamboo on the basis of comparative genomic analysis and RNA-Seq in Table 2.

**Table 2:** The genes of light-harvesting complex genes of photosystem I and II, and light-inducible genes in bamboo

Type	Locus Tag	Initial Annotation	Refined Annotation		Molecular Weight	Isoelectric Point	Amino Acids	Reciprocal best gene	
			Annotation	Synonym				*Oryza sativa* ^a^	*Arabidopsis* ^b^
LHC protein	PH01002452G0070	Chlorophyll a/b-binding protein	Light-harvesting complex II chlorophyll a/b binding protein 1 subunit 2	LHCB1.2	27941.94	5.1540	264	LOC_Os01g41710	AT2G34420
	PH01005133G0020	Chlorophyll a/b-binding protein	Light-harvesting complex II chlorophyll a/b binding protein 1 subunit 1	LHCB1.1	27801.90	5.1540	262	LOC_Os01g41710	AT2G34430
	PH01000046G0840	Chlorophyll a/b-binding protein	Light-harvesting complex II chlorophyll a/b binding protein 1 subunit 2	LHCB1.2	26038.72	5.0049	242	LOC_Os09g17740	AT2G34420
	PH01000653G0680	Chlorophyll a/b-binding protein	Light-harvesting complex II chlorophyll a/b binding protein 1 subunit 3	LHCB1.3	28081.26	5.4594	265	LOC_Os01g41710	AT1G29930
	PH01001378G0550	Chlorophyll a/b-binding protein	Light-harvesting complex II chlorophyll a/b binding protein 1	LHCB1.2	28107.30	5.2905	265	LOC_Os01g41710	AT2G34420
	PH01004107G0040	Chlorophyll a/b-binding protein	Light-harvesting complex II chlorophyll a/b binding protein 1 subunit 1	LHCB1.1	24516.04	8.5532	222	LOC_Os01g52240	AT2G34430
	PH01000120G1210	Chlorophyll a/b-binding protein	Light-harvesting complex I chlorophyll a/b binding protein 3	LHCA3	29564.98	7.8679	271	LOC_Os02g10390	AT1G61520
	PH01002466G0350	Chlorophyll a/b-binding protein	Light-harvesting complex I chlorophyll a/b binding protein 3	LHCA3	29512.88	7.8784	270	LOC_Os02g10390	AT1G61520
	PH01000173G0670	Chlorophyll a/b-binding protein	Light-harvesting complex I chlorophyll a/b binding protein 5	LHCA5	28131.71	6.7543	260	LOC_Os02g52650	AT1G45474
	PH01000184G0790	Chlorophyll a/b-binding protein	Light-harvesting complex II chlorophyll a/b binding protein 2 subunit 2	LHCB2.2	28531.46	5.6232	263	LOC_Os03g39610	AT2G05070
	PH01000848G0570	Chlorophyll a/b-binding protein	Light-harvesting complex II chlorophyll a/b binding protein 2 subunit 2	LHCB2.2	28502.42	5.4743	263	LOC_Os03g39610	AT2G05070
	PH01000625G0360	Chlorophyll a/b-binding protein	Light-harvesting complex II chlorophyll a/b binding protein 6	LHCB6	27233.50	8.7520	254	LOC_Os04g38410	AT1G15820
	PH01004502G0160	Chlorophyll a/b-binding protein	Light-harvesting complex II chlorophyll a/b binding protein 6	LHCB6	23571.17	8.8298	213	LOC_Os04g38410	AT1G15820
	PH01003036G0080	Chlorophyll a/b-binding protein	Light-harvesting complex I chlorophyll a/b binding protein 1	LHCA1	26527.45	6.2137	246	LOC_Os06g21590	AT3G54890
	PH01000198G1100	Chlorophyll a/b-binding protein	Light-harvesting complex II chlorophyll a/b binding protein 4	LHCB4	31873.19	5.3334	293	LOC_Os07g37240	AT5G01530
	PH01000198G0580	Chlorophyll a/b-binding protein	Light-harvesting complex II chlorophyll a/b binding protein 3	LHCB3	28704.91	5.6423	267	LOC_Os07g37550	AT5G54270
	PH01003394G0090	Chlorophyll a/b-binding protein	Light-harvesting complex II chlorophyll a/b binding protein 3	LHCB3	28784.91	5.2473	267	LOC_Os07g37550	AT5G54270
	PH01000086G1040	Chlorophyll a/b-binding protein	Light-harvesting complex I chlorophyll a/b binding protein 2	LHCA2	27826.79	5.6561	259	LOC_Os07g38960	AT3G61470
	PH01000008G1530	Chlorophyll a/b-binding protein	Light-harvesting complex I chlorophyll a/b binding protein 4	LHCA4	26759.65	6.5941	244	LOC_Os08g33820	AT3G47470
	PH01000177G0160	Chlorophyll a/b-binding protein	Light-harvesting complex I chlorophyll a/b binding protein 4	LHCA4	26941.80	6.5167	248	LOC_Os08g33820	AT3G47470
	PH01005293G0040	Chlorophyll a/b-binding protein	Light-harvesting complex I chlorophyll a/b binding protein 4	LHCA4	27144.08	7.8137	247	LOC_Os08g33820	AT3G47470
	PH01000242G0150	Chlorophyll a/b-binding protein	Light-harvesting complex II chlorophyll a/b binding protein 1 subunit 2	LHCB1.2	28137.19	5.1442	265	LOC_Os09g17740	AT2G34420
	PH01001205G0170	Chlorophyll a/b-binding protein	Light-harvesting complex II chlorophyll a/b binding protein 5	LHCB5	30184.53	5.4954	283	LOC_Os11g13890	AT4G10340
	PH01003298G0130	Chlorophyll a/b-binding protein	Light-harvesting complex II chlorophyll a/b binding protein 5	LHCB5	39648.83	6.5328	373	LOC_Os11g13890	AT4G10340
	PH01000262G1270	Chlorophyll a/b-binding protein	Light-harvesting complex II chlorophyll a/b binding protein 1 subunit 3	LHCB1.3	12744.85	9.2059	122	N.A.^c^	N.A.
	PH01001205G0190	Chlorophyll a/b-binding protein	N.A.	N.A.	18690.99	11.6682	177	N.A.	N.A.
	PH01002467G0130	Chlorophyll a/b-binding protein	chlorophyll a/b-binding protein	N.A.	18553.35	8.4212	171	N.A.	N.A.
	PH01000848G0670	Chlorophyll a/b-binding protein	Light-harvesting complex I chlorophyll a/b binding protein 5	LHCA5	21104.52	9.1401	189	N.A.	N.A.
	PH01000234G1250	Chlorophyll a/b-binding protein	chlorophyll a/b-binding protein		20461.82	9.3290	195	N.A.	N.A.
	PH01000947G0680	Chlorophyll a/b-binding protein	Light-harvesting complex II chlorophyll a/b binding protein 6	LHCB6	11791.44	5.2976	106	N.A.	N.A.
	PH01001974G0230	Chlorophyll a/b-binding protein	Light-harvesting complex I chlorophyll a/b binding protein 1	LHCA1	11230.86	7.6956	105	N.A.	N.A.
	PH01238153G0010	Chlorophyll a/b-binding protein	chlorophyll a/b-binding protein		13078.84	5.1662	115	LOC_Os07g37240.1	N.A.
	PH01000903G0290	Chlorophyll a/b-binding protein	Light-harvesting complex II chlorophyll a/b binding protein 7	LHCB7	18099.21	4.7718	167	LOC_Os09g12540.1	N.A.
	PH01000948G0030	Chlorophyll a/b-binding protein	chlorophyll a/b-binding protein	N.A.	13657.46	9.7935	119	N.A.	N.A.
	PH01003299G0020	Chlorophyll a/b-binding protein	Light-harvesting complex II chlorophyll a/b binding protein 1	LHCB1	12117.37	4.8291	112	N.A.	N.A.
LHC-like protein	PH01001858G0020	Early light-induced protein, chloroplast precursor	Early light-inducible protein 2	ELIP2	16699.45	11.4600	165	LOC_Os01g14410	N.A.
	PH01001936G0100	Early light-induced protein, chloroplast precursor	Early light-inducible protein 3	ELIP3	19715.93	11.8726	184	LOC_Os07g08160	N.A.
	PH01002764G0110	Early light-induced protein, chloroplast precursor	Early light-inducible protein 3	ELIP3	18684.59	7.8882	182	LOC_Os07g08160	N.A.
	PH01002764G0170	Early light-induced protein, chloroplast precursor	Early light-inducible protein 3	ELIP3	18616.46	6.2846	182	LOC_Os07g08160	N.A.
	PH01002445G0060	Expressed protein	One-helix protein 1	Ohp1	11717.95	10.5866	110	LOC_Os12g29570	N.A.
	PH01000097G0840	Expressed protein	Stress-enhanced protein 1	SEP1	11329.31	10.4135	111	LOC_Os10g25570	N.A.
	PH01000213G0560	Expressed protein	Stress-enhanced protein 1	SEP1	13876.24	11.4910	136	LOC_Os10g25570	N.A.
	PH01003491G0150	Expressed protein	Stress-enhanced protein 2	SEP2	19931.82	4.9638	188	LOC_Os04g54630	AT2G21970
	PH01000280G1190	Expressed protein	Stress-enhanced protein 3	SEP3	27405.31	5.2585	248	LOC_Os02g03330	N.A.
PsbS	PH01000004G0130	Chlorophyll a/b-binding protein	Non-photochemical quenching (NPQ) 4, Photosystem II subunit	PsbS	27916.47	7.8877	268	LOC_Os01g64960	N.A.
	PH01000293G0420	Chlorophyll a/b-binding protein	Non-photochemical quenching (NPQ) 4, Photosystem II subunit	PsbS	28036.82	8.9657	269	LOC_Os01g64960	AT1G44575
	PH01000845G0420	Chlorophyll a/b-binding protein	Non-photochemical quenching (NPQ) 4, Photosystem II subunit	PsbS	39164.92	5.9226	377	LOC_Os04g59440	N.A.

^a^ The annotation of bamboo and Reciprocal best genes with *O. sativa* were from BambooGDB

^b^ The annotation of bamboo and Reciprocal best genes with *A. thaliana* were from BambooGDB

^c^ N/A represent no available

In total, 42 genes in moso bamboo genome were annotated as *LHC* and *LHC*-like genes, including 38 *LHC* genes and 4 *ELIP* genes [11]. Here, we verified and refined 35, 9 and 3 genes as *LHC*, *LHC*-like, and *PsbS* genes in moso bamboo, respectively, based on (i) sequence analysis of reciprocal best gene with *A. thaliana* and *O. sativa*, (ii) secondary structure prediction, (iii) sequence motifs, (iv) domain search of KEGG, and (v) genome-wide transcriptome. For example, five genes without detailed annotation in moso bamboo genome were refined. The refined annotation of PH01002445G0060 was “one-helix protein 1”. The updated annotation of PH01000097G0840 and PH01000213G0560 was “stress-enhanced protein 1”. The refined annotation of PH01003491G0150 and PH01000280G1190 was “stress-enhanced protein 2” and “stress-enhanced protein 3”, respectively. Besides, three gene annotations, PH01000004G0130, PH01000293G0420, and PH01000845G0420, were updated to “non-photochemical quenching (NPQ) 4, photosystem II subunit” instead of initial annotation “chlorophyll a/b-binding protein”.

Notably, the initial annotation of PH01001205G0190 with “chlorophyll a/b-binding protein” maybe have problematic, not only because of the unavailable result in sequence comparative analysis of DNA and protein, such as nucleotide BLAST in nucleotide collection of NCBI and protein domain search, but also because of the unavailable expression value in this study. In addition, the expression value of PH01001205G0190 was also undetectable in some previous studies of moso bamboo transcriptome [8, 9, 17]. Thus, we suggested that there may be a mistake in the annotation of PH01001205G0190 initially, owing to the complexity of sequencing and assembling in moso bamboo.

There are 35 genes which encode for chlorophyll a/b-binding proteins in moso bamboo, higher than 23 genes in *A. thaliana* and 17 genes in *O. sativa*. Similarly, there are 12 genes which encode for LHC-like and PsbS in bamboo. Those in *A. thaliana* and *O. sativa* were 7 and 11, respectively. More copies of *LHC* genes indicated more energy may be required in the fast-growth stage of moso bamboo. The FPKM result indicated that the expression of major *LHC* genes was sequentially reduced with the increased light time. Meanwhile, the expression values of four *ELIP* genes appeared a large rise, consistent with the previous reports that ELIPs accumulated during early thylakoid development and light stress. In addition, the previous studies also confirmed that the primary function of LHC protein was the absorption of light through chlorophyll excitation and transfer of absorbed energy to photochemical reaction centers, while members of LHC-like and PsbS families were likely involved in stress protection [18–21].

### Genes related to reactive oxygen species in bamboo

Illumination of high light has possible trigging to overexcite the photosynthetic pigments and the electron transport chain [22]. When this exceeds the requirement of normal metabolism, it arises an excess of excitation energy in the photosystems. High energy states may be dissipated by NPQ and/or alternative processes (such as photorespiratory metabolism), and may be transferred to oxygen, thus generated toxic reactive oxygen species (ROS) [23]. To avoid damaging cellular components and even oxidative destruction of cells, ROS must be detoxified by ROS-scavenging pathway, which contained major enzymes, such as superoxide dismutase (SOD), ascorbate peroxidase (APX), catalase (Cat), glutathione peroxidase (GPX) and so on (Additional file 8). Based on bamboo annotation and the results of reciprocal best genes with *Arabidopsis* and *O. sativa*, we found a large number of ROS-scavenging enzymes in moso bamboo and their expressions were increased under high light, among which the maximum almost appeared in 8H, such as PH01000083G1490, PH01001010G0010, and PH01001942G0260.

Besides, the results of RNA-Seq data also depicted the average value of gene expression in Calvin cycle and photorespiratory metabolism was both declined under high light (Additional file 9). One possible reason was that CO_2_ diffusion, ATP synthesis and reluctant status, high light maybe negatively affect the Calvin cycle by reducing the content and activity of photosynthetic carbon reduction cycle enzymes. The limited CO_2_ assimilation, thus, leaded to the decreased gene expression in photorespiratory metabolism. Therefore, the levels of expressed genes in Calvin cycle and photorespiratory metabolism were suppressive under high light.

ROS signal transduction pathway fulfilled fundamental roles in ROS signal detecting, reception and delivering in order to regulate ROS-scavenging pathways. The results of DGEs analysis confirmed the genes in ROS signal transduction pathway were up-regulated under high light. However, the plant heat stress transcription factor (HSF) in the DGE dataset were more concentrated in 0.5H than 8H. High expressed genes, such as PH01000000G3800 and PH01000546G0840, were detected in 0.5H. These indicated HSF as one of ROS signals, maybe play essential roles in early stage of high light stress. In addition, the up-regulated genes annotated with HSP and HSP20/alpha crystalline family protein were detected as DGEs, such as PH01003771G0070, PH01000906G0020, PH01000967G0270 and so on, indicating they maybe associate with not only heat stress, but also ROS signal sensing.

Moreover, ROS signaling event was also associated with Ca^2+^ and Ca^2+^-binding proteins [24, 25], such as calmodulins. The up-regulated calmodulins were detected in 0.5H and 8H, and those in 0.5H were more than in 8H. Besides, a Ca^2+^ transporter, PH01000251G0960, was found as an up-regulated gene in 0.5H. Integrated with the previous results of redox-sensitive HSF and Ca^2+^, their signals maybe appear in preliminary stage of high light-induced, and some of their transporters may be involved in ROS signaling transduction in bamboo.

**Fig. 7:**
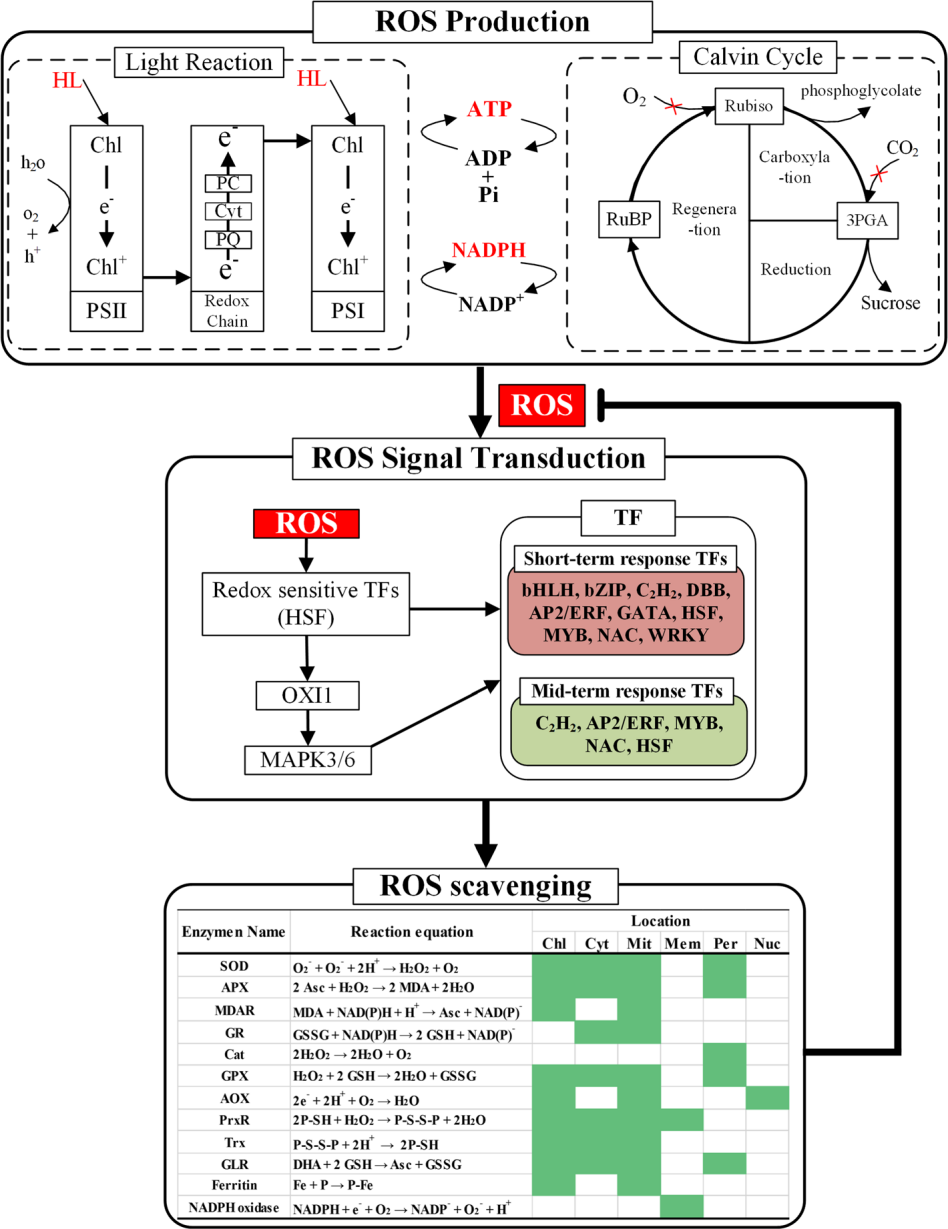
Generalized model of reactive oxygen species (ROS) pathway in bamboo. A large amount of high light (HL) produced an excess of excitation energy in the light reaction. Some of high energy maybe transferred to oxygen, thus generated toxic ROS. ROS can be detected by one of mechanisms, redox sensitive transcription factors. Phosphatidic acid and Ca^2+^ activate the serine/threonine protein kinase (OXI1). Then, the activation of OXI1 activated the mitogen-activated-protein kinase (MAPK) cascade (MAPK3/6) and these induced or activated short/mid-term response transcription factors that regulated the ROS-scavenging and related pathways. In the ROS-scavenging, main ROS enzymes, reaction equation and reaction location were listed in moso bamboo. Abbreviations: HL, high light; PSII, photosystem II; PSI, photosystem I; Chl, chlorophyll; PC, plastocyanin; Cyt, cytochrome; PQ, plastoquinone; 3PGA, 3-phosphoglycerate; RuBP, ribulose-1,5-biphosphate; ROS, reactive oxygen species; SOD, superoxide dismutase; APX, ascorbate peroxidase; MDAR, monodehydroascorbate reductase; GR, glutathione reductase; Cat, catalase; GPX, glutathione peroxidase; AOX, alternative oxidase; PrxR, peroxiredoxin; Trx, thioredoxins; GLR, glutaredoxin; chl, chloroplast; cyt, cytosol; mem, membrane; mit, mitochondria; nuc, nuclei; per, peroxisomes

As one of significant ROS sensing, serine/threonine protein kinase (OXI1) was reported previously [26, 27], which played a central role in the activation of mitogen-activated-protein kinase (MAPK) 3 and 6 associated with Ca^2+^. In this study, the up-regulated OXI1 genes, such as PH01000015G0230, PH01000016G0280 and PH01001215G0410, were found in 0.5H and 8H, suggesting OXI1 maybe play a key role in ROS signal transmission of bamboo under high light. As controlling the activation of different TFs associated with various defense mechanisms in response to ROS stress, the MAPK3/6 was not enlisted in DEG output, but the FPKM of MAPK3/6 was higher expression in 0.5H and 8H, and the maximum mainly appeared in 0.5H, which maybe depict MAPK3/6 signaling was strengthened in early stage of high light treatment. Taken together, as a crucial network of ROS signal transduction, including redox-sensitive HSF, Ca^2+^, OXI1, MAPK3/6 and some TFs, this pathway (Fig. 7) was activated under high light and the peak signal was appeared in the initial stage. As another ROS signal pathway, the phosphatases was suppressed by ROS, then inhibited phosphatases promoted the expression of OXI1 and MAPK3/6 [28]. Subsequently, MAPK3/6 activated many TFs participated in ROS-scavenging. Some down-regulated phosphatase genes concentrated in 8H, such as lipid phosphatase gene (PH01000297G0870), HAD superfamily phosphatase gene (PH01001136G0170), and phosphate transporter gene (PH01000381G0230). Therefore, the phosphatases in ROS signal pathway maybe play a considerable role in ROS signal transferring under high light treatment for a relatively long time.

### Potential roles of TFs in regulating ROS

To protect cells and sustain growth under high light, bamboo responded to unfavorable changes in their environments through developmental, physiological and biochemical ways. These responses required some genes expressed in response to light stress, which were regulated by a network of transcript factors (TFs) [23]. In the ROS signal networks, TFs played critical roles in response to high light stress though regulating the gene expression, by which TF was capable of binding the *cis*-acting elements present in the promoter of a target gene.

As previous studies indicated, many TFs maybe involve in ROS signal networks under high light stress. Firstly, HSF, as one of key regulators in heat shock response, will up-regulate heat shock proteins (HSPs) [29]. HSPs not only can be activated and expressed during normal conditions, such as the cell growth and developments, but also can be induced by some stresses, such as heat shock, infection and heavy metals [30]. Secondly, NAC was associated with the induction of genes related to flavonoid biosynthesis, leading to the accumulation of anthocyanin in response to high light stress [31–41]. Thirdly, MYB played important roles in both stomatal and non-stomatal responses by the regulation of stomatal numbers and sizes, and metabolic components, respectively, in the regulation of photosynthetic and related metabolism [42, 43]. Fourthly, AP2/ERF was a large family of plant-specific TFs that regulated the expression of abiotic stress responsive gene. Finally, WRKY, as one of plant-specific TFs, contained one large family of regulatory protein in plants [44–46], which participated in various biotic stress response and several developmental and physiological processes [47–52]. Some WRKYs in *A. thaliana* were significantly enhanced by H_2_O_2_, which was one specific ROS [53–60]. These indicated WRKY maybe perform an important role under oxidative stress.

Therefore, combined with the previous studies and RNA-Seq data, the results illustrated many bamboo TFs, such as HSF, MYB, bZIP, AR2/ERF, NAC, and WRKY, maybe also involve in ROS signal networks under high light (Table 3) and play crucial roles in regulating, acclimating, and modulating gene expression in photosynthesis process in response to high light. Besides, based on the analysis of expression data and DGEs, the TFs of NAC, WRKY, AR2/ERF, and bHLH might fulfill important roles in short-term (0.5H), while those of C_2_H_2_, HSF, bZIP, and MYB might perform vital roles both in short-term (0.5H) and mid-term (8H) in response to high light.

**Table 3:** Transcript factors maybe involve in response to high light stress in moso bamboo

TF family	Differentially expressed genes			TF list
	Samples	Up/down-regulated	Number	
bHLH	0.5H *vs*. CK	Up	11	PH01000179G0630,PH01000260G1030,PH01000323G0290,PH01000783G0460,PH01001228G0390,PH01001641G0420,PH01002972G0210,PH01002972G0240,PH01003790G0150,PH01005158G0070,PH01103680G0010
	8H *vs*. CK	Down	3	PH01000105G0060,PH01000110G0500, PH01000331G0630
bZIP	0.5H *vs*. CK	Up	1	PH01000242G0910
	8H *vs*. CK	Down	2	PH01000105G1660,PH01000145G1240
C_2_H_2_	0.5H *vs*. CK	Up	10	PH01000040G0510,PH01001038G0420,PH01001038G0430,PH01001065G0460,PH01001535G0570,PH01001743G0120,PH01001743G0130,PH01002187G0020,PH01002335G0340,PH01005966G0040
	0.5H *vs*. CK	Down	2	PH01000140G0830,PH01000021G0290
	8H *vs*. CK	Up	2	PH01000054G1270,PH01000673G0600
AP2/ERF	0.5H *vs*. CK	Up	22	PH01000046G1730,PH01000084G1170,PH01000129G0360,PH01000131G1230,PH01000131G1240,PH01000343G0330,PH01000437G0390,PH01000543G0370,PH01000559G0630,PH01000573G0670,PH01000668G0390,PH01000841G0400,PH01000842G0220,PH01000890G0360,PH01001360G0530,PH01001480G0410,PH01000034G0340,PH01002169G0130,PH01002648G0300,PH01002733G0270,PH01003475G0200,PH01007432G0010
	8H *vs*. CK	Up	2	PH01000016G1360,PH01000018G0790
	8H *vs*. CK	Down	2	PH01000038G0910,PH01001704G0270
HSF	0.5H *vs*. CK	Up	7	PH01000081G0140,PH01000174G0590,PH01000314G0470,PH01000546G0840,PH01002606G0040,PH01003169G0070,PH01000000G3800
	8H *vs*. CK	UP	1	PH01000701G0030
MYB	0.5H *vs*. CK	Up	14	PH01000004G1580,PH01000043G2100,PH01000169G1060,PH01000302G0910,PH01000309G0470,PH01000415G0090,PH01000515G0560,PH01002987G0150,PH01000617G0820,PH01000688G0030,PH01000912G0430,PH01001208G0070,PH01001287G0090,PH01001996G0370
	0.5H *vs*. CK	Down	3	PH01007341G0010,PH01002611G0010,PH01007341G0010
	8H *vs*. CK	Up	2	PH01003809G0130,PH01003918G0100
	8H *vs*. CK	Down	3	PH01000604G0860,PH01000383G0320,PH01000931G0030
NAC	0.5H *vs*. CK	Up	11	PH01000004G1630,PH01000053G1650,PH01000112G0050,PH01000122G1000,PH01000261G0890,PH01000309G0360,PH01000877G0160,PH01001177G0140,PH01001669G0130,PH01001843G0210,PH01002777G0100
	8H *vs*. CK	Down	1	PH01003138G0210
WRKY	0.5H *vs*. CK	Up	17	PH01000046G1680,PH01000112G1290,PH01000182G0790,PH01000534G0230,PH01000659G0050,PH01000735G0110,PH01001037G0370,PH01001737G0080,PH01001777G0070,PH01002011G0380,PH01002018G0330,PH01002744G0230 PH01000009G2100,PH01002800G0110,PH01003110G0050,PH01003922G0080,PH01004940G0100
	8H *vs*. CK	Down	3	PH01003579G0140,PH01000823G0340,PH01002022G0200

## Conclusions

A global view of gene expression profiles and a large-scale stage-specific transcriptome profile in leaves of moso bamboo provided more accurate insights into the gene and gene regulation in response to high light based on deeply sequencing technology. In total, 1,293 genes were identified as differentially expressed genes and 47 gene annotations for LHC protein superfamily members in moso bamboo were refined. In addition, the pathway of ROS, including ROS signal transduction and ROS-scavenging, was detected. Meanwhile, 171 genes involved in ROS-scavenging were identified. Besides, some essential expressed genes and transcript factors were found, which played crucial roles in different regulated processes under high light. These results may provide a key resource for further experimental research on function of some proteins involved in light stress, and expand our knowledge of the mechanisms in bamboo under light stress.

## Methods

### Plant materials and high light treatment

Moso bamboo (*Phyllostachys edulis*) seedlings were potted in our laboratory under long-day conditions (16 h light/8 h dark) at 25 °C, with a light intensity of 200 μmol · m^−2^ · s^−1^. The air relative humidity was about 50 %. For high light stress, one-year-old seedlings were moved from normal light condition (200 μmol · m^−2^ · s^−1^) to high light (1200 μmol · m^−2^ · s^−1^) provided by cool white fluorescent tubes. The third leaf on the top of seedlings were selected for the measurement of chlorophyll fluorescence parameters after 0 h, 0.5 h, 1 h, 2 h, 4 h, 8 h and 12 h high light treatments, respectively.

### Measurement of chlorophyll fluorescence parameters

*In vivo* chlorophyll fluorescence parameters of leaves from one-year-old seedling of moso bamboo were measured with Dual PAM-100 fluorometer (Walz, Effeltrich, Germany). The following parameters were calculated: the maximum quantum yield of PSII *F*_v_/*F*_m_ = (*F*_m_-*F*_o_)/*F*_m_ and the non-photochemical quenching of NPQ was calculated as (*F*_m_ –*F*_m_’)/ *F*_m_’[10], where *F*_o_ is the minimum fluorescence in the dark-adapted state, *F*_m_ and *F*_m_’ are the darkness-adapted and light-adapted maximum fluorescence upon illumination of pulse (0.6 s) of saturating light, respectively. *F*_o_ and *F*_m_ were determined after 20 min dark adaptation. Each parameter was measured with ten replicates per treatment. All data were statistically analyzed using SPSS software.

### RNA isolation, cDNA library construction, and RNA sequencing

Of the previous materials, three samples of moso bamboo, containing the leaves under high light (1200 μmol · m^−2^ · s^−1^) for 0 h (CK), 0.5 h (0.5H), and 8 h (8H) were collected, respectively. Each sample was collected from at least three individual bamboos randomly selected in genetically distinct, and the mixed bundle was quickly frozen in liquid nitrogen for RNA isolation. The total RNA was isolated from samples of all selected bamboo tissues using TRIZOL Reagent Solution (Invitrogen, Carlsbad, CA, USA) on the basis of the manufacturer’s instructions. The extracted RNA was treated with RNase-free DNase I for 30 min at 37 °C in order to remove the residual DNA. The quality and quantity of RNA were detected using a NanoDrop 2000 spectrophotometer. Reverse transcription was conducted with Reverse Transcription System (Promage, USA) [61]. The cDNA library construction and normalization were performed as previously described [62]. Then the pooled libraries were sequenced by the Illumina HiSeq^TM^ 2000 platform (Illumina, San Diego, CA, USA).

### Bioinformatics analysis

Firstly, adaptor sequences and low quality sequences were trimmed using Trimmomatic [62]. Secondly, to accurate align clean reads to the reference bamboo genome and explore unannotated gene, insert size of cDNA libraries and *de novo* assembly of the clean reads were performed by Trinity software [63]. Then, as the reference genome, the genome sequences and annotation of moso bamboo (version 1) was downloaded from Bamboo Genome Database (www.bamboogdb.org) [11]. The filtered sequences were mapped to the reference bamboo genome using TopHat2 [64]. Subsequently, the aligned read files were processed by Cufflinks [65]. After reads were assembled into transcripts, their abundance was estimated and normalized using the number s of reads per kilobase of exon sequence in a gene per million mapped reads [66]. In the analysis of functional and structural annotation, GO enrichment was carried out using Ontologizer [67].

### Primer design and validation of RNA-Seq data

The primer pairs for flanking sequences of each unique gene were designed automatically using the Primer3 (Additional file 10). All primers were tested with rTaq (TaKaRa, Japan) before quantitative real time PCR (qRT-PCR) performed. The qRT-PCR reactions were performed on Qtower (analyticjena, Germany) with Roche LightCycler 480 SYBR Green I Master Kit. The reaction volume was 10 μL and contained 5.0 μL 2 × SYBR Green I Master Mix, 0.8 μL cDNA, 0.2 μL forward primer and reverse primer each (5 μM), and 3.8 μL ddH_2_O. All reactions were repeated three times. The qRT-PCR procedure consisted of 95 °C for 10 min and 50 cycles of 95 °C for 10 s, 60 °C for 10 s. For each condition, the qRT-PCR experiments were performed as biological triplicates. The relative gene expression level was calculated with the 2^-△△Ct^ method [68] using *NTB* as the reference gene [69].

### Accession numbers

All sequence data for three samples from this article have been deposited in the Short Read Archive (SRA) at the NCBI database under the following accession numbers: SRR2035212, SRR2035263, and SRR2035327.

## Additional files

Additional file 1:
**Differential samples isolated from moso bamboo for RNA-Seq analysis.** (XLSX 10 kb) Additional file 2:
**Relative expression values of RNA-Seq and qRT-PCR in selected 17 genes.** (XLSX 12 kb) Additional file 3:
**The list of expressed genes with FPKM ≥1 in Venn diagram.** (XLSX 289 kb) Additional file 4:
**Distribution of gene expression values among samples.** (XLSX 10 kb) Additional file 5:
**The significant GO terms in within-sample.** (XLSX 20 kb) Additional file 6:
**The list of genes and significant GO terms in five groups based on clustering affinity search technique.** (XLSX 293 kb) Additional file 7:
**The list of differentially expressed genes in three samples.** (XLSX 135 kb) Additional file 8:
**Gene and expression of the reactive oxygen species scavenging in moso bamboo.** (XLSX 29 kb) Additional file 9:
**The values of gene expression in Calvin cycle and photorespiratory metabolism.** (XLSX 12 kb) Additional file 10:
**Primers of 17 selected genes from moso bamboo utilized in qRT-PCR.** (XLSX 11 kb)

## Abbreviations

APX: ascorbate peroxidase
BLAST: basic local alignment search tool
CAST: clustering affinity search technique
Cat: catalase
DEG: differentially expressed gene
ELIPs: early light-inducible proteins
FPKM: fragments per kilobase of gene per million mapped fragments
GO: gene ontology
GPX: glutathione peroxidase
HSF: heat stress transcription factor
KEGG: kyoto encyclopedia of genes and genomes
LHC: light-harvesting chlorophyll a/b-binding
MAPK: mitogen-activated-protein kinase
NPQ: non-photochemical quenching
OHPs: one-helix light-inducible proteins
PsbS: photosystem II subunit S
qRT-PCR: quantitative real time PCR
RNA-Seq: RNA sequencing
ROS: reactive oxygen species
SEPs: stress-enhanced proteins
SOD: superoxide dismutase
TF: transcript factor
